# Transcriptomic and targeted metabolome analyses revealed the regulatory mechanisms of the synthesis of bioactive compounds in *Citrus grandis* ‘tomentosa’

**DOI:** 10.7717/peerj.16881

**Published:** 2024-02-23

**Authors:** Xinmin Huang, Xiaoli Liu, Qi Wang, Yanqing Zhou, Shiting Deng, Qinqin He, Hanbing Han

**Affiliations:** 1 College of Biology and Food Engineering, Guangdong University of Petrochemical Technology, Maoming, China; 2 Guangdong Provincial Engineering Technique Research Center for Exocarpium Citri Grandis Planting and Processing, Maoming, China; 3 Maoming Branch, Guangdong Laboratory for Lingnan Modern Agriculture, Maoming, China

**Keywords:** Citrus grandis ‘tomentosa’, Transcriptomic, Targeted metabolome, Synthesis of bioactive compounds

## Abstract

Exocarpium Citri Grandis is a popular Chinese herbal medicine prepared from* Citrus grandis* ‘tomentosa’, and it is rich in several bioactive compounds, including flavonoids, coumarins, and volatile oils. However, studies are yet to elucidate the mechanisms of synthesis and regulation of these active components. Therefore, the present study examined the profiles of flavonoids and volatile oil bioactive compounds in plant petals, fruits, and tender leaves, and then performed RNA sequencing on different tissues to identify putative genes involved in the synthesis of bioactive compounds. The results show that the naringin, naringenin, and coumarin contents of the fruitlets were significantly higher than those of the tender leaves and petals, whereas the tender leaves had significantly higher levels of rhoifolin and apigenin. A total of 49 volatile oils, of which 10 were mainly found in flowers, 15 were mainly found in fruits, and 18 were mainly found in leaves, were identified. RNA sequencing identified 9,942 genes that were differentially expressed in different tissues. Further analysis showed that 20, 15, and 74 differentially expressed genes were involved in regulating flavonoid synthesis, regulating coumarin synthesis, and synthesis and regulation of terpenoids, respectively. CHI1 (Cg7g005600) and 1,2Rhat gene (Cg1g023820) may be involved in the regulation of naringin synthesis in *C. grandis* fruits. The HDR (Cg8g006150) gene, HMGS gene (Cg5g009630) and GGPS (Cg1g003650) may be involved in the regulation and synthesis of volatile oils in *C. grandis* petals. Overall, the findings of the present study enhance our understanding of the regulatory mechanisms of secondary metabolites in *C. grandis*, which could promote the breeding of *C. grandis* with desired characteristics.

## Introduction

Exocarpium Citri Grandis (ECG), prepared from *Citrus grandis* ‘tomentosa’, is a popular traditional Chinese medicine (TCM), which is used to treat cold and cough (Jiang et al., 2014). *C. grandis* ‘tomentosa’ has been cultivated in Huazhou City, Guangdong Province, for over 1500 years for medical purposes. Modern medical research has shown that the primary active components in ECG are volatile oil, flavonoids, and polysaccharides (Xie et al., 2013; Su et al., 2020). ECG was listed in a TCM formula in the Guidelines for the Diagnosis and Treatment of Coronavirus Disease 2019 (6th edition) in China, and studies have shown that combined treatment with TCM is effective in the management of critically ill COVID-19 patients (Zhang et al., 2020).

Among the bioactive contents of ECG, naringin is the main chemical component used to assess the quality standard of ECG. Naringin is a flavonoid with several biological activities, including expectorant, antitussive, and anti-asthmatic activities (Bodas et al., 2011; Luo et al., 2012; Zhu et al., 2019). Studies have shown that naringin plays an expectorant role by promoting the movement of tracheal cilia, inhibiting the overexpression and secretion of MUC5AC and MUC5B mucin, and preventing the proliferation of airway goblet cells (Lin et al., 2008). Additionally, a previous study found that naringin extracted from ECG exhibited an anti-inflammatory effect by inhibiting lipopolysaccharide-induced pulmonary edema and myeloperoxidase and inducible nitric oxide synthase activities in mice (Liu et al., 2011). Moreover, naringin extracted from orange can reduce the levels of interleukin-8, leukotriene B4, and tumor necrosis factor in bronchoalveolar lavage and duodenal fluids, and significantly alleviate cough symptoms in guinea pigs exposed to cigarette smoke (Nie et al., 2012; Peng et al., 2019; Zhu et al., 2019). Furthermore, naringin is effective against severe acute respiratory syndrome (Su et al., 2004). Another important flavonoid in orange is rhoifolin, which has been shown to possess antioxidative, antihypertensive, and anti-tachycardia properties (Rao et al., 2011). Moreover, some studies have shown that rhoifolin, a bioactive component in orange, can improve diabetes-related complications and inhibit the growth of cancer cells (Eldahshan & Azab, 2012; Eldahshan, 2013).

Over the years, the volatile compound profile of ECG has been examined using a combination of different extraction methods, including steam disintegration (SD), headspace solid phase microextraction (HS-SPME), and solvent extraction (SE), and chromatography methods, including gas chromatography mass spectroscopy. Overall, a total of 77, 56, and 48 compounds have been identified in extracts obtained *via* SD, HS-SPME, and SE methods, respectively (Xie et al., 2013). Limonene, *α*-pinene, and myrcene are the major essential oil components, with limonene accounting for up to 70% of the essential oil content of ECG (Li et al., 2009). Studies have shown that limonene possesses anti-inflammatory and expectorant effects (Wang, Tang & Bidigare, 2005), whereas *α*-pinene possesses antitumor and antioxidant effects (Chen et al., 2014).

The quality and efficacy of ECG for medicinal use is dependent on its bioactive compound profile, indicating that the adoption of methods for increasing the accumulation of secondary metabolites could improve ECG efficacy. An analysis of the appearance and secondary metabolites of the three main varieties of *C. grandis* revealed that the trichomes and flavonoids of the three varieties were different (Fan et al., 2019; Tian, Fan & Zeng, 2021). Fan et al. (2023) further screened 25 genes and 16 transcription regulatory factors related to flavonoid biosynthesis using metabolomics and transcriptomics, which may be involved in the biosynthesis of flavonoids in *C. grandis* ‘tomentosa’ fruit. Further analysis of the role of Jumonji C (JMJC) domain family members in the regulation of the external morphology and internal quality of *C. grandis* shows that some CgJMJC genes show different expression patterns during the fruit development of three varieties, indicating that they may play a role in the formation of unique phenotypes (Tian, Fan & Zeng, 2021); however, their specific functions and whether they regulate the synthesis of secondary metabolites still need further study. In addition, studies have shown that CgPBA1, Ca^2+^-dependent nuclease, and Zn^2+^-dependent nuclease are involved in the programmed cell death that leads to secretory cavity formation in *C. grandis* fruits (Bai et al., 2020; Huai et al., 2021; Liang, Bai & Wu, 2021).

Zheng et al. (2023) constructed a regulatory pathway for *C. grandis* flavonoids and identified a transcription factor CgMYB108, which can negatively regulate the flavonoid synthesis pathway by interacting with the promoters of PAL (phenylalanine ammonia lyase) and FNS (flavone synthesis). However, although there have been studies on the synthesis and regulation mechanisms of some active ingredients in citrus red, the main focus has been on the synthesis of flavonoids, and there have been no research reports on the synthesis and regulation of volatile oils (Fan et al., 2023; Zheng et al., 2023). Therefore, the aim of the present study was to apply transcriptomics and targeted metabolomics techniques to detect differences in the contents and gene expressions of five flavonoids and volatile oils in different tissues of *C. grandis*, and further analyze the synthesis and regulation mechanisms of bioactive compounds. The outcome of the present study could provide insights on the regulatory mechanisms of bioactive compounds in *C. grandis* ‘tomentosa’, which could serve as a theoretical basis for further research and breeding of *C. grandis* ‘tomentosa’ with specific qualities.

## Materials & Methods

### Materials

The ‘Huanglong’ variety of *C. grandis* ‘tomentosa’ was sampled from the planting base of Xiangxiu Huajuhong Industrial Co., Ltd (Linchen Town, Huazhou City, 22°49′11″N, 110°36′11″E). The fruits (F, 30-day-old), petals (P, just blooming), and tender leaves (L, 20-day-old) were sampled on March 2, 2018. The plant materials, including 10 fruits, 20 leaves, and 50 flowers, were obtained each of nine 10-year-old individuals, , and the samples of three individuals were obtained as biological repeats. Three biological replicates were collected from the tissues, frozen rapidly with liquid nitrogen, and stored at −80 °C for further analysis.

### Extraction and determination of the flavonoids and coumarin contents of *C. grandis*

The extraction and determination of the flavonoid and coumarin contents of *C. grandis* were performed using a method developed by Han et al. (2014). Samples of the different tissues were dried to a constant weight in an oven at 60 °C (based on the traditional method), crushed, and sieved through a 40 µm mesh. The powder was used for extraction. For the extraction, 0.25 g of flower, fruit, and leaf powder samples, respectively, were put into a 25 ml extraction tube. Then, 25 ml of 60% ethanol solution was added and the mixture was well shaken in an ultrasonic water bath at 40 °C for 30 min. Thereafter, the mixture was centrifuged at 6,000 rpm for 10 min. Qualitative and quantitative analysis of four types of flavonoids in the supernatants were performed using high-performance liquid chromatography (HPLC), using a SPD-20a HPLC system (Shimadzu Corporation, Kyoto, Japan) coupled with a thermo ODS chromatographic column (250 mm × 4.6 mm, 5 µm) and a Lc-20at UV detector. Mobile phase A comprised pure methanol, whereas mobile phase B consisted of 1% glacial acetic acid aqueous solution. The detection conditions were as follows: gradient elution (0–10 min, 35% a; 10–30 min, 35% a–65% a; 30–35 min, 65% a; 35–42 min, 65% a–35% a); a flow rate of 1.0 ml/min; a column temperature of 40 °C; and an injection volume of 10 µL. Naringin and naringenin were detected at 283 nm, while rhoifolin, apigenin, and coumarin were detected at 340 nm. Quantitative analysis of the bioactive compounds was performed based on the peak area of the standard curve of each compound. Naringin standard (no.110722-200810) was provided by the China Institute for the control of pharmaceutical and biological products, while naringin (no.120824), rhoifolin (no.120711), and apigenin (no.120906) standards were provided by Sichuan Vicki Biotechnology Co., Ltd. All standards used for HPLC were of high purity (≥98%). The naringin, rhoifolin, naringenin, apigenin, and coumarin contents of ECG were recorded as the average values of three biological repetitions.

### Extraction and determination of the volatile compounds in *C. grandis*

Approximately 0.1 g of flower, fruit, and leaf powder samples were extracted using 10 mL of absolute alcohol at 30 °C for 30 min, followed by centrifugation. The supernatant was dehydrated using anhydrous sodium sulfate at 4 °C for 24 h. The mixture was then centrifuged at 8000 rpm for 10 min and then filtered through a 0.22 µm filter membrane. The volatile organic compound profile of the supernatant was determined by on-line gel permeation chromatography-gas chromatography-mass spectrometry (Han et al., 2018). Principal Components Analysis (PCA) and heatmap analysis and Venn diagram construction were performed using the omicshare (http://www.omicshare.com/tools) online tools.

### Extraction and high-throughput sequencing of RNA from *C. grandis*

Total RNA was extracted from the tissues using the RNeasy plant Mini Kit (Qiagen, Valencia, CA, USA) according to the manufacturer’s instructions. RNA was treated with RNase-free DNase I (Takara Bio, Ostu, Japan) to remove residual DNA. RNA quantity and integrity were assessed using electrophoresis (1% agarose gel) and an Agilent 2100 Bioanalyzer (Agilent Technologies, Santa Clara, CA, USA), respectively. The mRNA was enriched and purified using oligo magnetic beads and then broken into 200 NT short fragments using a fragmentation buffer. Thereafter, first strand cDNA was synthesized using mRNA as the template. The second strand was synthesized immediately. The QiaQuick PCR extraction kit (Qiagen, Venlo, Holland) was used to purify the cDNA fragment, repair base ends, and connect the Illumina sequencing adapters. The size of the ligated product was determined using agarose gel electrophoresis. Finally, the product was amplified using PCR (Wang et al., 2016) and sequenced on an Illumina Hiseq 4000 (Illumina, San Diego, CA, USA) platform. The sequencing length was double ended 150 bp. All sequence data were deposited at the Genome Sequence Archive of the Beijing Institute of Genomics Data Centerunder accessions CRA006910.

### Transcriptome data analysis: annotation and differential expression analysis

First, the raw reads were processed to remove joint sequence, low-quality reads (the base number of an alkali with mass value Q ≤ 20 accounts for more than 50% of the whole read) and reads with N ratio greater than 10%. The read was compared to the ribosomal database to remove rRNAs and obtain high-quality reads using bowtie v.2.2.8 software (Langmead et al., 2009). Thereafter, the clear reads were aligned against *C. grandis* genome version 1 (http://citrus.hzau.edu.cn/download.php) using Tophat2 v.2.1.1 software (Kim et al., 2013; Wang et al., 2017). The genomic reads were assembled by comparing them using Cufflinks (Trapnell et al., 2010), and then the assembly results of multiple samples were combined using cuffmerge. Transcripts with assembly errors were filtered to generate a unique annotation file for subsequent analysis. Genes with size ≥ 200 bp and exon ≥ 2 were mapped and functionally annotated to NCBI RefSeq, Kyoto Encyclopedia of Genes and Genomes (KEGG), and Swissprot databases.

The expressed values of all genes were calculated and normalized to fragments per kilobase of transcript per million fragments mapped (FPKM). Differential expression analysis of genes between two samples was performed using the DESeq package in R software (R Core Team, 2021). Genes with false discovery rate <0.05 and fold change —log2— ≥ 1 were considered differentially expressed genes (DEGs) between samples. Pearson’s correlation analysis was performed to determine the relationship between two samples, which was visually display in the form of a heatmap. Transcriptome power analysis using functions in RNASeqPower (Corley et al., 2017)

### Gene ontology and KEGG pathway enrichment analysis

The identified DEGs were aligned against gene ontology (GO) and KEGG databases for functional annotation and pathway analysis, using KOBAS (http://kobas.cbi.pku.edu.cn/) and omicshare (http://www.omicshare.com/tools) online tools, respectively.

### Quantitative reverse transcription PCR

To verify the RNA-seq results, nine genes were selected for quantitative reverse transcription PCR (qRT-PCR) analysis using *Actin* as the internal reference gene (Tian, Fan & Zeng, 2021). The reactions were performed on Bio-Rad CFX96 Touch (Bio-Rad, CA, USA) using 5 µL of 2 × ChamQ Universal SYBR qPCR Master Mix (Vazyme Biotech Co., Ltd., Nanjing, China) containing 1.5 µL of cDNA template, 0.4 µL of each primer (10 µmol/ µL), and 2.7 µL of nuclease-free water. PCR conditions and Melting curve analyses are described by Huang et al. (2017). Relative gene expression levels were normalized to that of *Actin* and determined using the 2^−ΔΔCT^ method (Livak & Schmittgen, 2001). All reactions were performed in triplicate.

### Data analysis

All data are presented as the mean of several values. Differences in the bioactive contents between the plant tissues were determined using Duncan’s new compound polar difference method and SigmaPlot v.11 software (Systat Software Inc., CA, USA). Means were considered statistically significant at *p* < 0.05.

## Results

### Flavonoid and coumarin contents of different tissues of *C. grandis*

The naringin (418.32 mg/g), naringenin (2.73 mg/g), and coumarin (0.42 mg/g) contents of the tender fruits were significantly higher than those of the tender leaves and petals, whereas the tender leaves had significantly higher levels of rhoifolin (57.70 mg/g) and apigenin (2.41 mg/g). Specifically, the naringin content of the tender fruits was more than six times those of the tender leaves and petals, whereas the rhoifolin content of the young leaves was more than 10 times those of the other two tissues (Table 1, Table S1).

**Table 1:** Flavonoid and coumarin contents of different tissues (petal, fruit, leaf) of *Citrus grandis* ‘tomentosa’.

	Naringin (mg/g)	Rhoifolin (mg/g)	Naringenin (mg/g)	Apigenin (mg/g)	Coumarin (mg/g)
Petal	63.40 ± 3.51b	3.59 ± 0.79b	0.34 ± 0.03c	0.75 ± 0.15b	0.12 ± 0.01b
Fruit	418.32 ± 6.83a	5.34 ± 1.54b	2.73 ± 0.39a	0.40 ± 0.06b	0.42 ± 0.03a
Leaf	61.24 ± 1.07b	57.70 ± 2.75a	0.98 ± 0.03b	2.41 ± 0.34a	0.02 ± 0.00c

**Notes.**

Values with different lowercase letters indicate significant differences at *p* <0.05 according to Duncan’s multiple- range tests.

### Volatile oil content of different tissues of *C. grandis*

**Figure 1:**
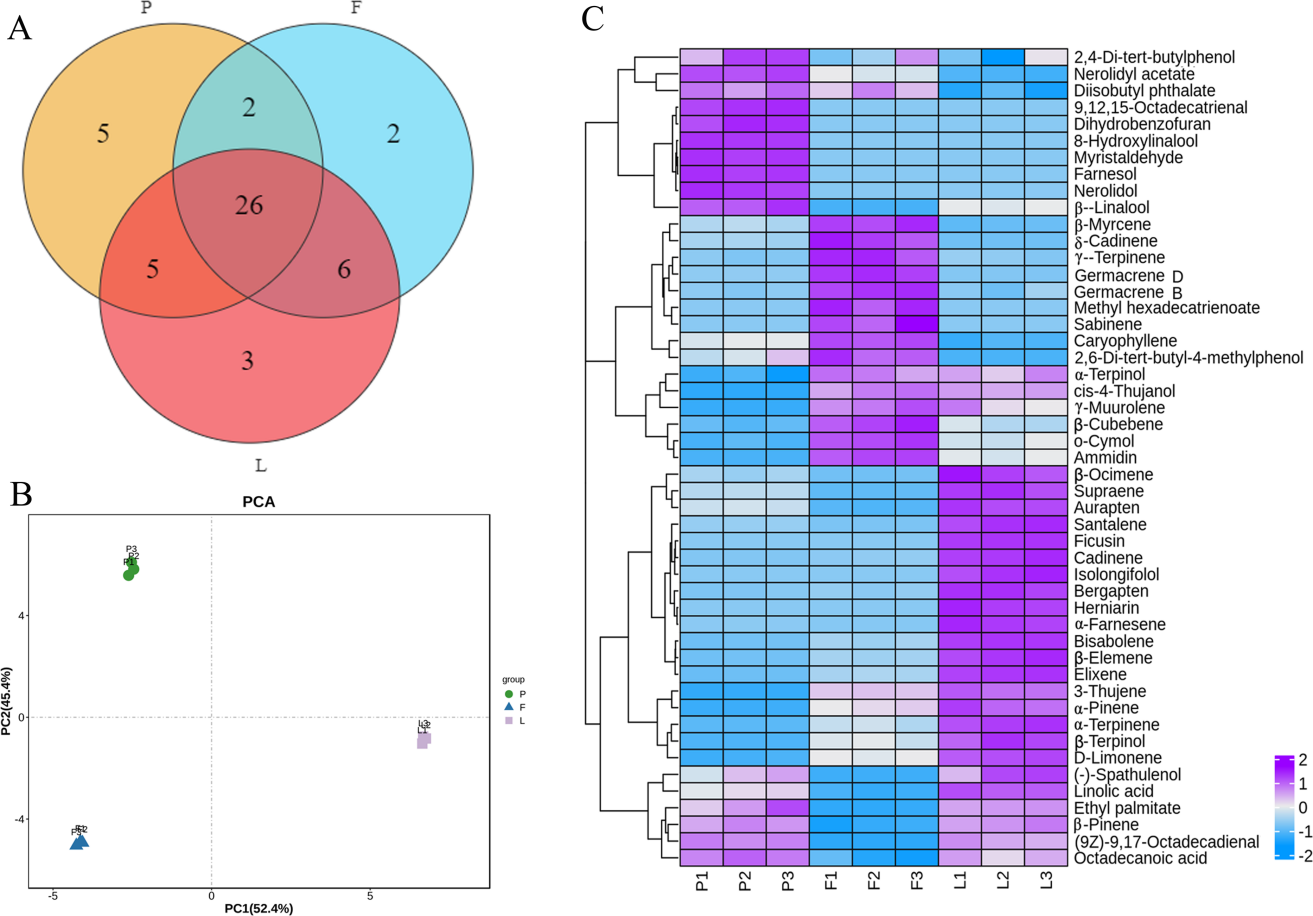
Venn diagram, principal components analysis (PCA) and heatmap analysis and of volatile compounds in different tissues of *C. grandis*. (A) Venn diagram; (B) principal components analysis (PCA); (C) Heatmap.

A total of 49 volatile compounds were identified in the tender leaves, fruits, and petals of ECG, among which 15 were monoterpenes, 16 were sesquiterpenes, one was triterpene, and 17 were fatty acids (Table S2). Additionally, 26 volatile compounds were common to the petals, fruitlets, and tender leaves, three were found only in the leaves, two only in the fruits and five only in the petals (Fig. 1A). PCA results showed that the three biological replicates from different tissues were well clustered, indicating good reproducibility. The good separation of different organizations indicates that there were differences in the compositions of volatile oils among different organizations (Fig. 1B). Moreover, among 38 volatile compounds identified the petals, the main compounds were *β*-pinene (7.53%), *β*-myrcene (15.32%), *γ*-terpinene (8.23%), nerolidol (20.57%), and farnesol (14.85%). Among 36 volatile compounds identified in the fruit, the main compounds were *β*-myrcene (24.35%), *γ*-Terpinene (20.70%), and germacrene D (18.46%). Furthermore, a total of 40 components were identified in the leaves, among which the main components were *β*-pinene (8.27%), *β*-myrcene (10.31%), *γ*-terpinene (8.76%), ficusin (8.52%), and squalene (9.25%) (Table S2). Furthermore, heat map analysis results showed that the volatile oil contents in different tissues could be divided into four categories. The first category mainly exists in the petals, such as 2,4-Di tert butylphenol and Nerolidyl acetate. The second category mainly exists in flowers, such as Myrcene. The third category mainly exists in leaves, such as a-Ocimene, and the fourth category is distributed in petals and leaves, such as 3-Thujene (Fig. 1C).

### Sequence analysis of transcriptome in different tissues

**Figure 2:**
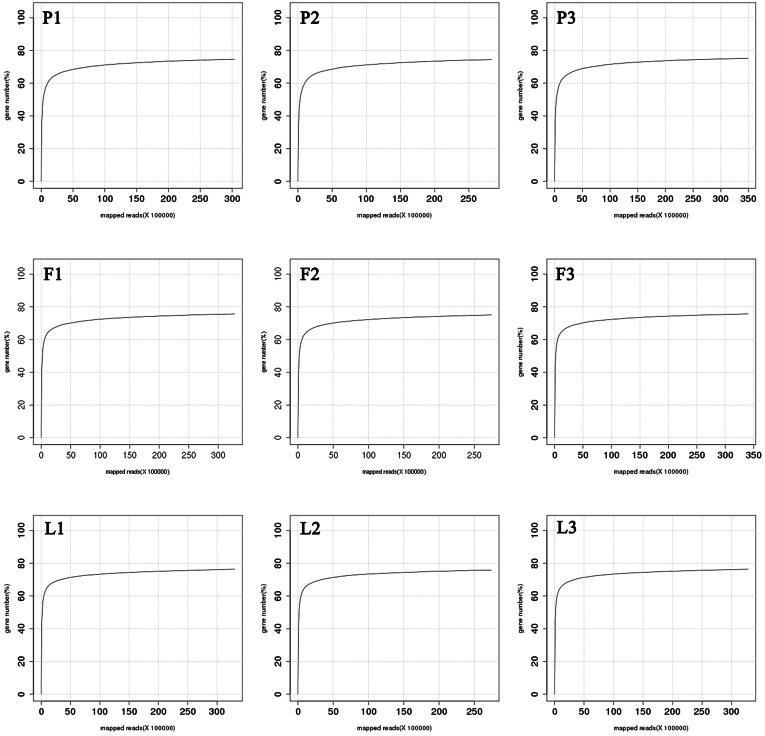
RNA-seq sequencing saturation graph of nine samples.

The amount of transcriptome sequencing data of per sample was 6 G, and the saturation analysis showed that per sample had reached saturation (Fig. 2). A total of 55.02 G HQ clean data were obtained from nine samples (Table 2), following the removal of low-quality reads. The Q30 values (*i.e.,* the sequencing accuracy was 99.9%) were >95.0%. The percentages of high-quality reads were >96%, indicating that the sequencing quality was high. Subsequently, the clean reads were aligned against the pomelo genome (version 1), of which an average of 86.83% of reads from nine libraries were successfully mapped to the grapefruit genome. The statistical power of this experimental design, calculated in RNASeqPower, is 0.76% (Table S3). A total of 31,215 genes were obtained, among which 21,672 were known genes in the genome and 1,092 were novel genes (Table 2).

**Table 2:** Summary statistics for *Citrus grandis* ‘tomentosa’ clean reads mapped to the reference genome.

Sample	HQ clean data (bp)	Q30 (%)	Clean reads num	HQ clean reads (%)	Removed rRNA (%)	Mapped reads (%)	Known gene Num (%)	Novel transcripts Num	All gene Num
F1	6496980422	95.69	45570432	97.44	99.13	87.18	18745 (62.23%)	985	19730
F2	5487688552	95.16	38653888	97.27	98.44	86.83	18549 (61.58%)	986	19535
F3	6777069746	95.47	47689364	97.15	98.85	87.3	18783 (62.35%)	1004	19787
L1	6536357902	95.61	46082620	97.12	99.03	87.41	19049 (63.24%)	1038	20087
L2	5431951224	95.28	38720492	96.39	99.01	87.51	18872 (62.65%)	1015	19887
L3	6463830697	95.40	45742412	96.86	99.18	87.63	19016 (63.13%)	1026	20042
P1	5744442040	95.63	40369808	97.24	99.38%	89.28	18443 (61.23%)	990	19433
P2	5419369397	95.56	38170656	97.14	98.34%	88.95	18411 (61.12%)	987	19398
P3	6661105287	95.57	46953410	97.09	98.92%	88.97	18682 (62.02%)	1004	19686

**Figure 3:**
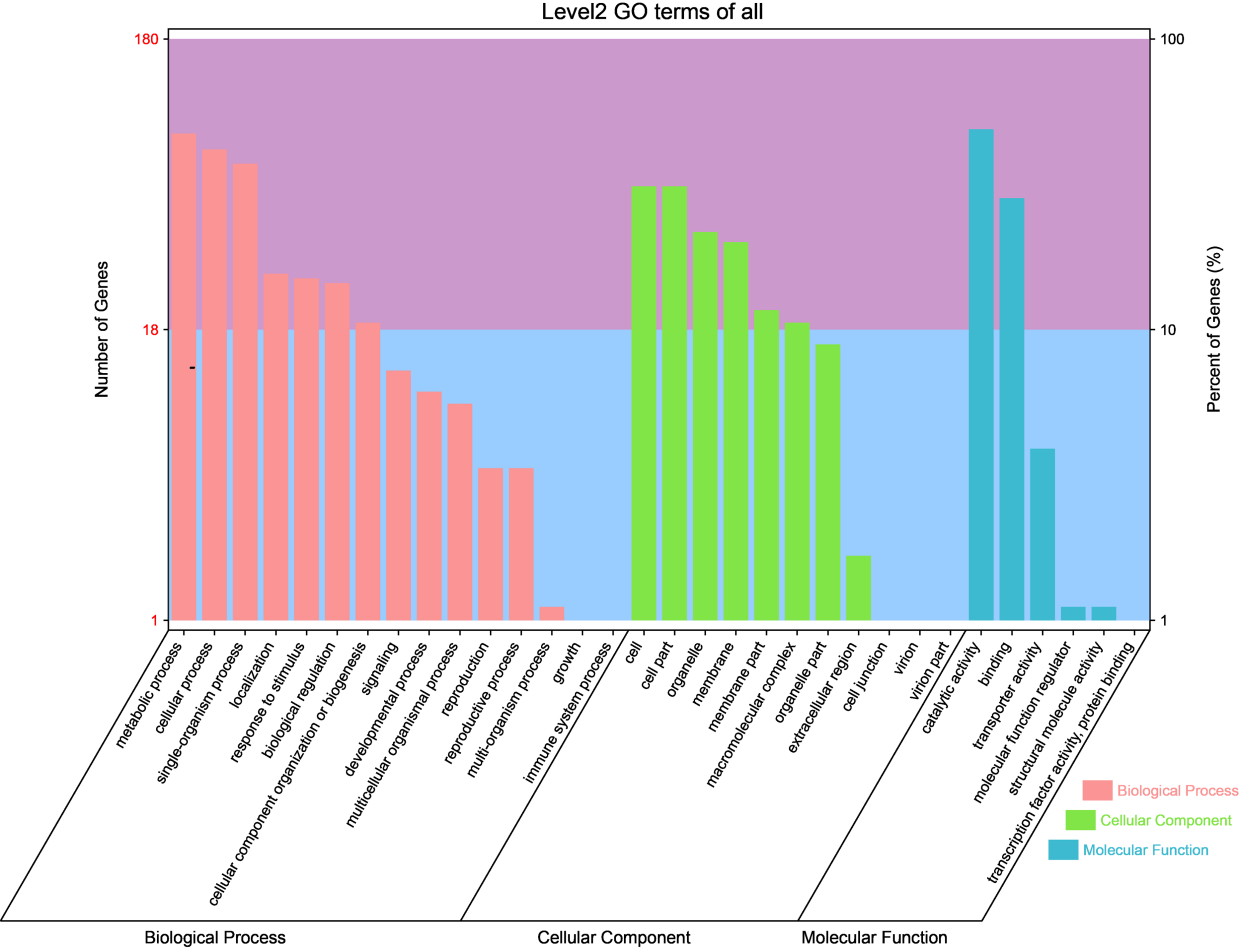
Gene ontology classification of novel transcripts in different tissues (petal, fruit, leaf) of *Citrus grandis* ‘tomentosa’.

GO analysis of the novel genes showed that a total of 180 new transcripts were annotated in 15 terms in “biological processes,” 11 terms in “cellular components,” and six terms in “molecular functions” (Fig. 3). Specifically, 47.2, 41.7, and 37.2% of the genes were annotated in “metallic process,” “cellular process,” and “single organization process,” respectively, in ‘biological processes.’ Additionally, 31.1, 31.1, and 21.7% of the genes were annotated in “cell part,” “cell,” and “organelle,” respectively, in ‘cellular components.’ Moreover, 48.9% and 28.3% of the genes were annotated in “catalytic activity” and “binding” in ‘molecular functions.’

### Analysis of sequence repeatability of the transcriptome in different tissues

**Figure 4:**
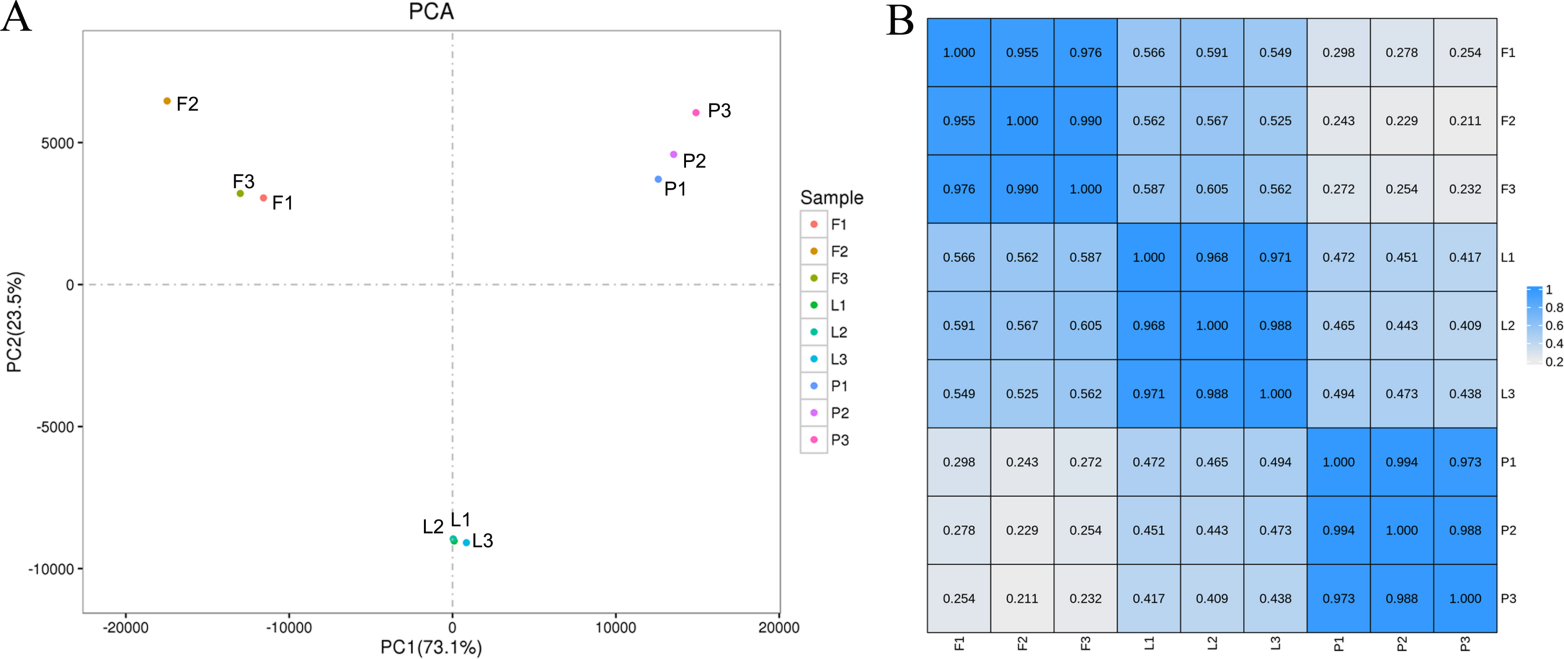
Principal component analysis (PCA) score plot and correlation between gene expression levels in different tissues (petal, fruit, leaf) of *Citrus grandis* ‘tomentosa’. (A) PCA score plot; (B) heatmap of correlation. A Pearson’s correlation coefficient (r) > 0.8 was considered as the significance cutoff.

Principal component analysis (PCA) was performed to visualize the gene expression profiles of samples from the different tissues of ECG. The results show that the three biological replicates from each tissue clustered together, indicating that the sequencing repeatability was high. Additionally, the gene expression profiles of the leaves formed a distinct cluster in the second principal component PC2, whereas the gene expression profiles of the fruitlets formed distinct clusters in the first principal component PC1, indicating that there are significant differences in gene expression in different tissues (Fig. 4A). Furthermore, Pearson’s correlation analysis was performed to determine the relationships between the expression profiles of different tissues, using the FPKM values of the genes (R^2^ > 0.8 indicates a significant correlation between the two samples). The results show that the Pearson correlation coefficient among three replicates of the same tissue was greater than 0.95 (Fig. 4B), indicating that the sequencing had high repeatability and could be used for subsequent differential expression analysis.

**Figure 5:**
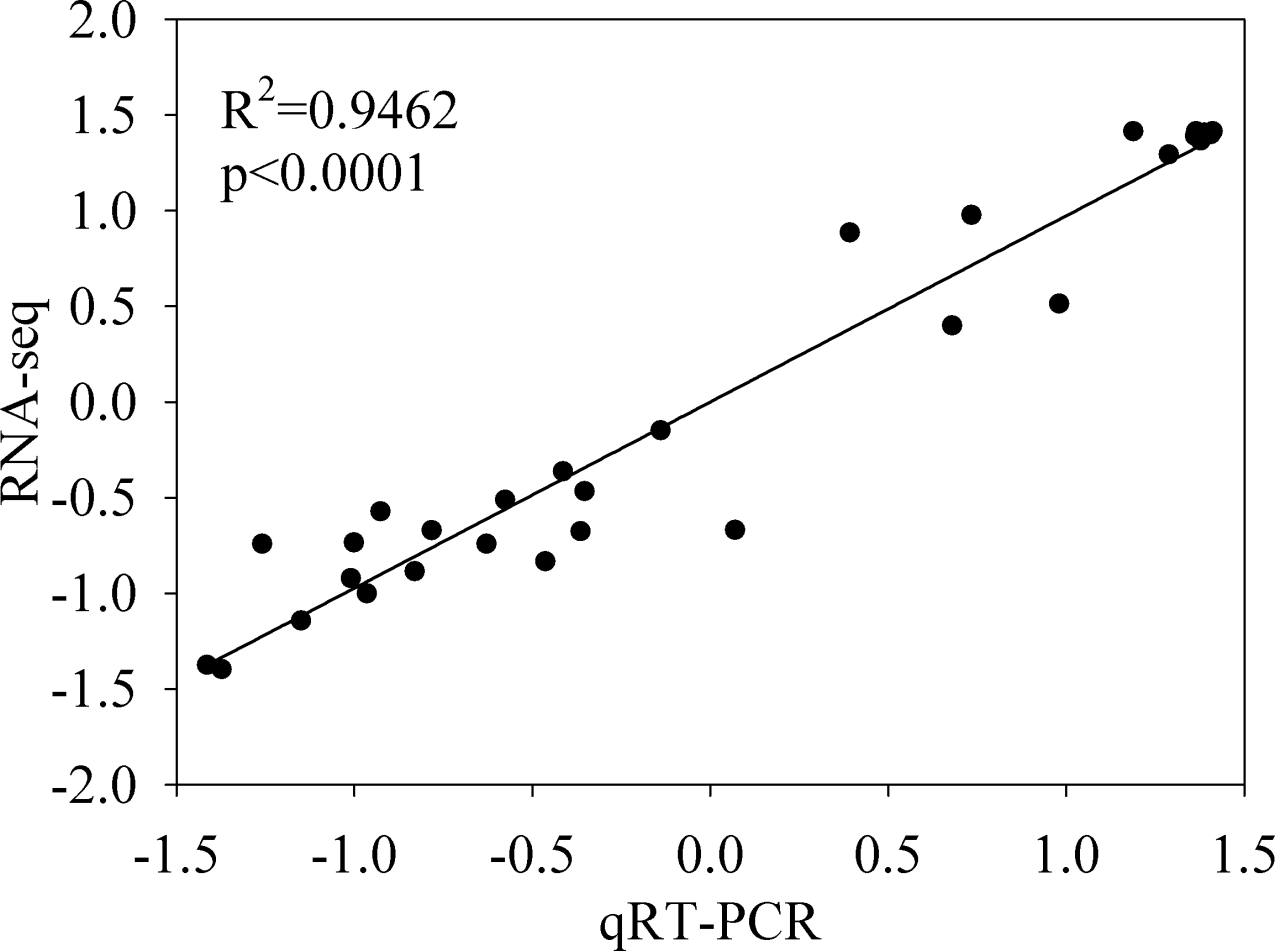
Validation of the RNA-seq gene expression data by quantitative reverse transcription PCR (qRT-PCR). Twelve differentially expressed genes from the RNA-seq data were used for qRT-PCR assays. Pearson’s correlation coefficients between the RNA-seq and qRT-PCR data were significant, with (r) > 0.8 as the significance threshold. The row Z-scores of the fragments per kilobase of transcript per million fragments mapped and qRT-PCR data are shown.

The expression profiles of twelve genes were amplified using RT-qPCR to confirm the accuracy and reproducibility of the RNA-seq results, using specific primers (Table S4). The results of the RT-qPCR and RNA-seq show similar expression profiles for the twelve genes (Table S5). Additionally, the Pearson’s correlation coefficient between the RNA-Seq and RT-qPCR data was 0.9462 (*P* < 0.0001), indicating the reliability of the RNA-Seq data (Fig. 5).

### Analysis of gene differential expression in different tissues of *C. grandis*

**Figure 6:**
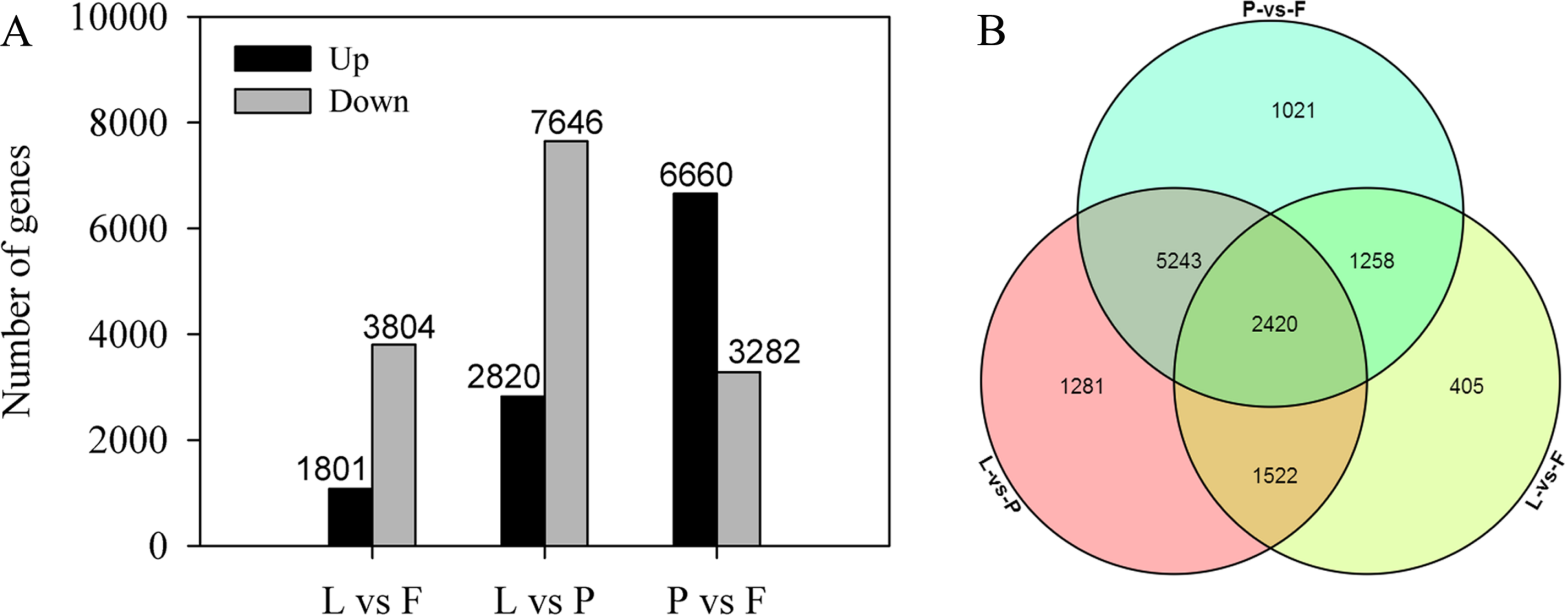
Differentially expressed genes (DEGs) in different comparisons between the tissues (petal, fruit, leaf) of *Citrus grandis* ‘tomentosa’. (A) Pairwise comparisons of gene expression. The number of up- and down-regulated genes in each pair is presented. (B) Venn diagram of the number of DEGs in the three tissues. The number of genes is presented.

Differential expression analysis was performed to identify DEGs between the three tissues. A total of 11,514 DEGs were identified in a pairwise comparison of three tissues, among which 5,605, 10,466, and 9,942 DEGs were identified in the L *vs* F, L *vs* P, and P *vs* F comparison groups, respectively. Additionally, 1,081 and 3,804 genes, 2,820 and 7,646 genes, and 6,660 and 3,282 genes were upregulated and downregulated in the L *vs* F, L *vs* P, and P *vs* F comparison groups, respectively (Fig. 6A). Among the differential genes, 2,420 genes were differentially expressed in the three tissues, 405 were differentially expressed only between tender fruits and leaves, 1,281 genes were differentially expressed only between petals and leaves, and 1,021 genes were differentially expressed only between tender fruits and petals (Fig. 6B).

### Functional analysis of differentially expressed genes

**Figure 7:**
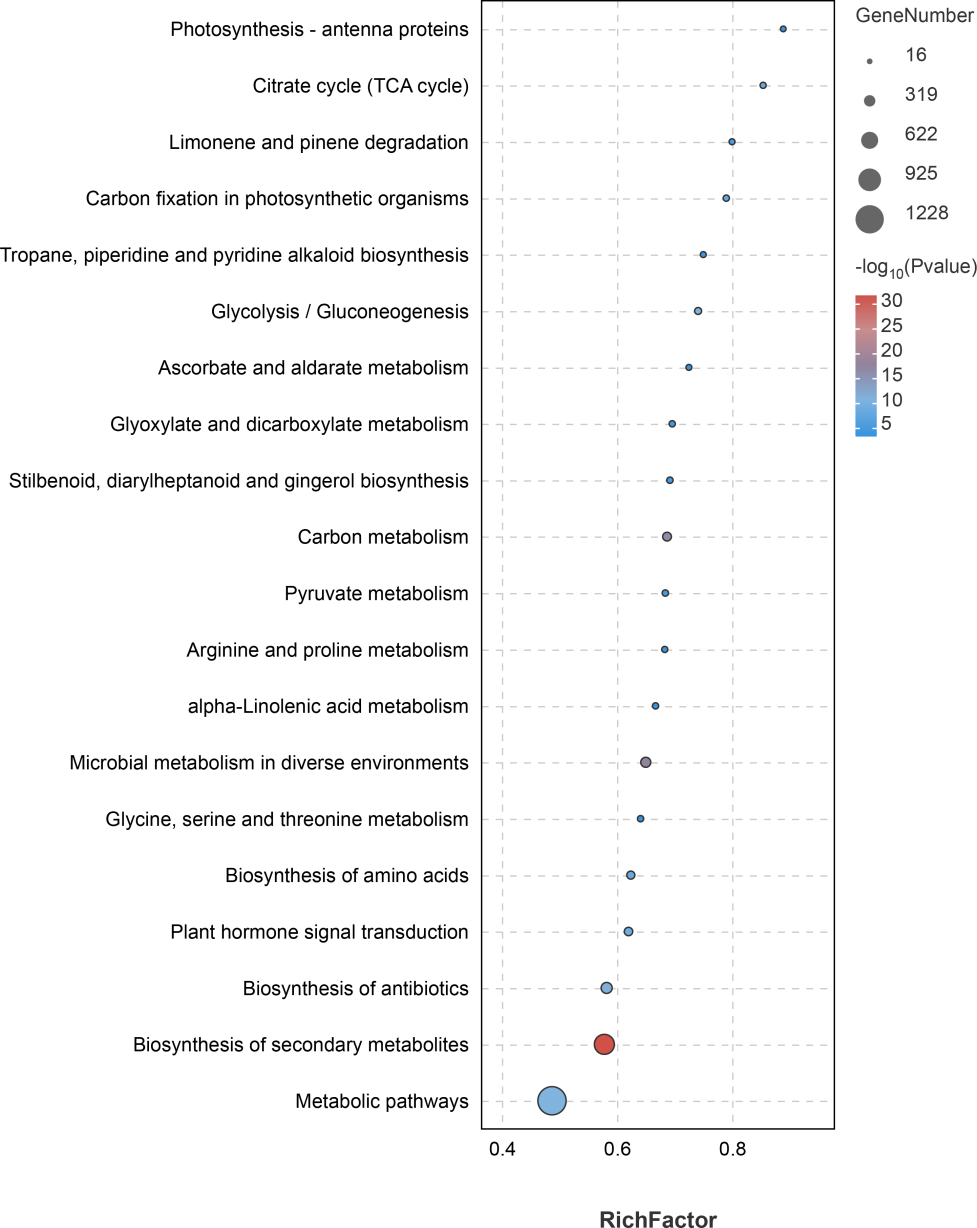
Top 20 significantly enriched pathways by differentially expressed genes identified in the tissues (petal, fruit, leaf) of *Citrus grandis* ‘tomentosa’.

KEGG pathway analysis of the DEGs showed that a total of 2,566 DEGs were significantly enriched in 43 KEGG pathways, among which “metabolic pathways” (1,228 DEGs, 47.86%) and “biosynthesis of secondary metabolites” (787 DEGs, 30.67%) were the most significantly enriched pathways (Fig. 7, Table S6). In addition, the pathways involved in flavonoid synthesis, such as “Flavonoid biosynthesis” (46 DEGs, 1.80%), “Flavone and Flavonol biosynthesis” (six DEGs, 0.23%), as well as pathways involved in the synthesis of volatile oil compounds, such as “Dieterpenoid biosynthesis” (32 DEGs, 1.24%), “Monoterpenoid biosynthesis” (19 DEGs, 0.74%), and “Fatty acid metabolism” (43 DEGs, 1.68%), were enriched significantly (Fig. 7, Table S6). The results indicate that the DEGs are mainly involved in the biosynthesis of flavonoids and volatile oils.

### Gene expression of flavonoids and coumarin synthesis pathway

**Figure 8:**
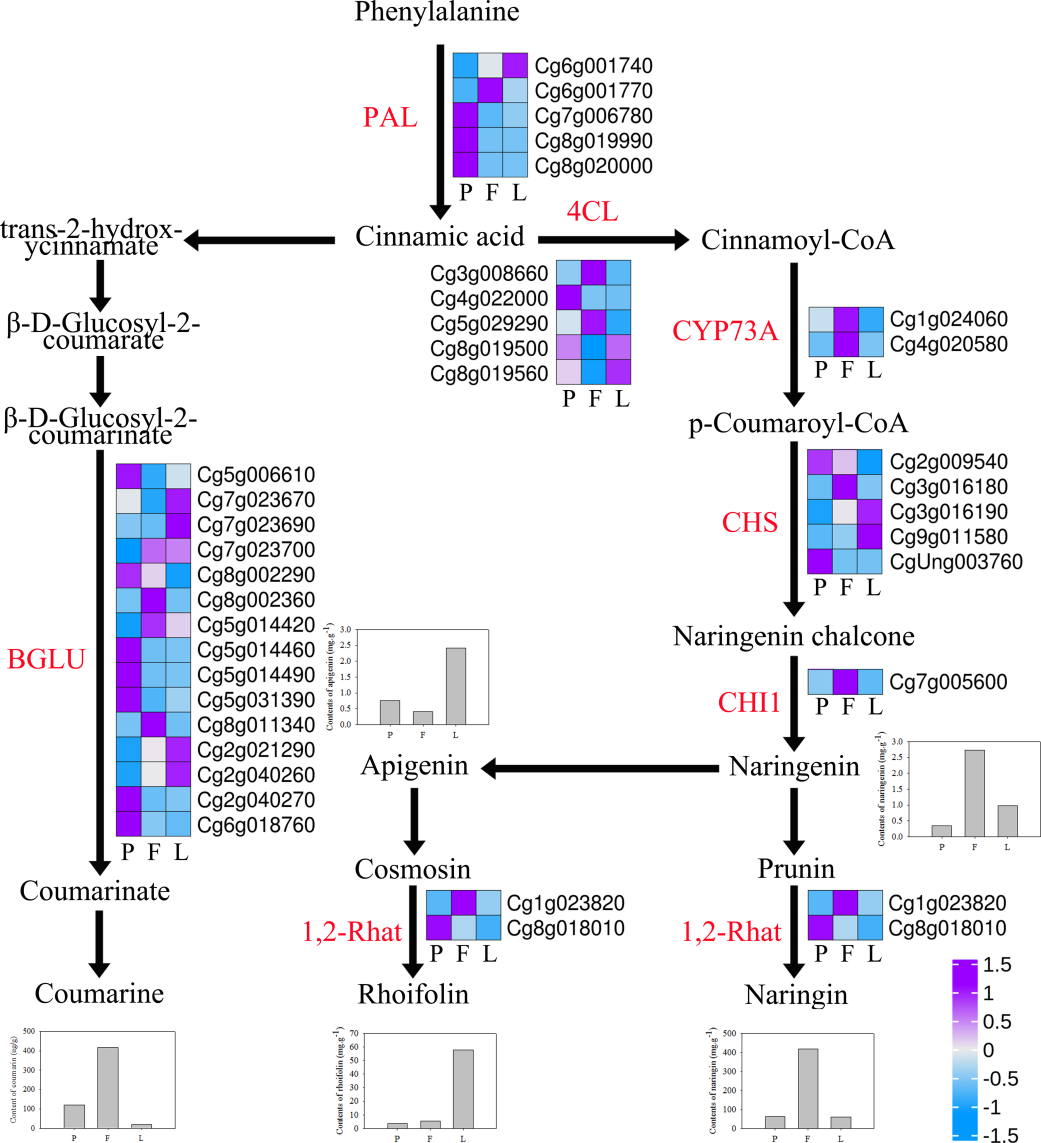
Expression profiles of flavonoids and coumarin synthesis-related genes in *Citrus grandis* ‘tomentosa’. PAL, phenylalanine ammonia-lyase; 4CL, 4-coumarate-CoA ligase; CYP73A, trans-cinnamate 4-monooxygenase; CHS, chalcone synthase; CHI1: chalcone isomerase; C12RT1, 1-¿ 2 UDP-rhamnosyltransferase; BGLU, *β*-glucosidase. The bar graph represents the change of material. The heatmap shows the trend of gene expression. The values represent the Z-scores for the fragments per kilobase of transcript per million fragments mapped data.

The expression profiles of genes related to naringin, rhoifolin, naringenin, and apigenin synthesis were examined in the different tissues of ECG (Fig. 8, Table S7). The results show that five phenylalanine enzyme (PAL) genes, which are involved in phenylpropane biosynthesis, were differentially expressed in the three different tissues, with the petals having the highest expression of three of the genes. Additionally, five 4CL genes regulating cinnamic acid production of cinnamoyl-COA were differentially expressed in the three tissues, among which two were upregulated in the fruitlets. Moreover, two CYP73A genes involved in regulating the synthesis of p-coumarinyl-COA were significantly upregulated in the fruitlets. Furthermore, five chalcone synthase (CHS) genes involved in regulating the production of grapefruit ligand chalcone were differentially expressed in the three tissues. One CHI1 gene involved in regulating naringenin synthesis was significantly upregulated in the fruitlets, which is evidenced by the higher naringenin content of the fruitlets. Moreover, naringenin is a precursor of apigenin, which in turn is a precursor of rhoifolin. Additionally, the fruitlets had higher expression levels of one 1,2Rhat gene (Cg1g023820), which is involved in the synthesis of naringin. Furthermore, 15 BGLU genes, which are involved in coumarin synthesis, were differentially expressed between the three tissues, with the fruitlets having significantly higher expression of two BGLU genes (Fig. 8, Table S7).

### Gene expression of the terpenoids synthesis pathway

**Figure 9:**
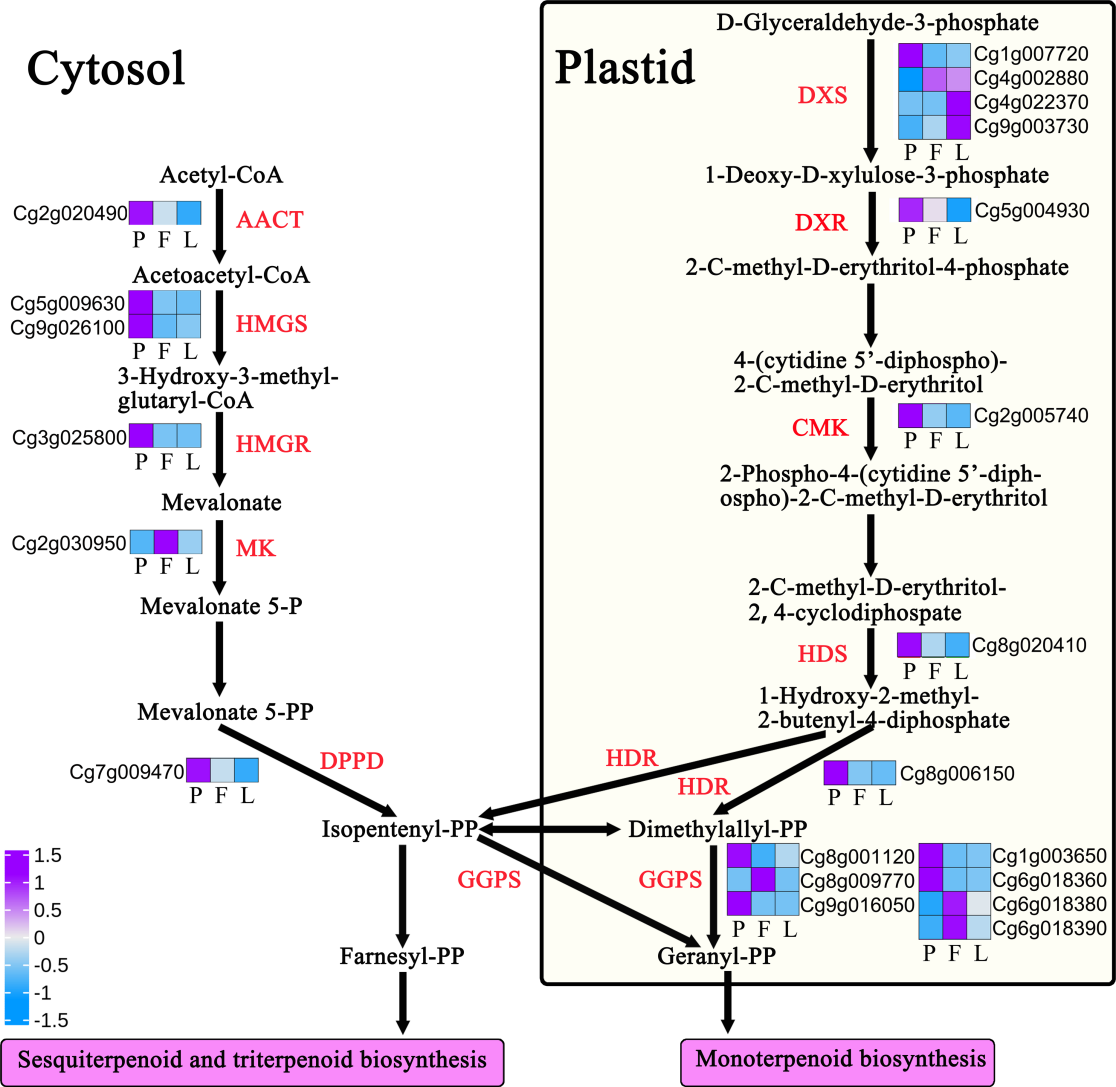
Expression profiles of terpenoid backbone biosynthesis-related genes in *Citrus grandis* ‘tomentosa’. AACT, acetyl CoA thiolase; HMGS, 3-hydroxy-3-methylglutaryl CoA synthase; HMGR, hydroxymethylglutaryl CoA reductase; MK, mevalonate kinase; DPPD: diphosphomevalonate decarboxylase; DXS, 1-deoxy-d-xylose-5-phosphate synthase; DXR, 4-hydroxy-3-methylbut-2-en-1-yl diphosphate synthase; CMK, cytidine 4-diphosphate-2-c-methyl-d-erythritose kinase; HDS, 1-hydroxy-2-methyl-2-(E)-butenyl-4-diphosphate synthase; HDR, 1-hydroxy-2-methyl-2-(E)-butenyl-4-pyrophosphate reductase; GGPS, geranylgeranyl pyrophosphate synthase. The heatmap shows the trend of gene expression; the values represent the Z-scores for the fragments per kilobase of transcript per million fragments mapped data.

The biosynthesis of terpenoids comes from the main chain formed by the cytosol and plastid pathways, and further forms a series of substances such as monoterpenes, sesquiterpenes, and diterpenes. This process involves many key enzymes. A total of 29 DEGs of encoding key enzymes are involved in the regulation of terpenoid backbone biosynthesis, including acetyl CoA thiolase (AACT), 3-hydroxy-3-methylglutaryl CoA synthase (HMGS), hydroxymethylglutaryl CoA reductase, mevalonate kinase, mevalonate 5-diphosphate decarboxylase, 1-deoxy-d-xylose-5-phosphate synthase, cytidine 4-diphosphate-2-c-methyl-d-erythritose kinase (CMK), 2-c-methyl-d-erythritol-2,4-cyclophosphate synthase, 1-hydroxy-2-methyl-2-(E)-butenyl-4-diphosphate synthase (HDS), and 1-hydroxy-2-methyl-2-(E)-butenyl-4-pyrophosphate reductase (HDR) (Table S8, Fig. 9). Further analysis showed that 19 encoding key enzyme genes were differentially expressed in the monoterpene biosynthesis pathway, while 26 encoding key enzyme genes were differentially expressed in the sesquiterpene and triterpene biosynthesis pathway (Table S8, Fig. 9).

## Discussion

*C. grandis* was first recorded in 1765 AD, and it is the raw material for the ECG TCM material. It has been used to treat respiratory diseases for hundreds of years (Su et al., 2020). Recent studies have shown that ECG possess antioxidant, anticancer, antibacterial, and anti-inflammatory activities owing to its rich bioactive compound profile (Fan et al., 2019; Peng et al., 2019; Liu et al., 2022). Duan et al. (2014) examined the bioactive compound profile of different tissues of *C. grandis* and identified the presence of naringin and rhoifolin in the fruit and flower. Similarly, the findings of the study showed that the fruitlets had significantly higher concentrations of naringin, naringenin, and coumarin, while tender leaves had significantly higher concentrations of rhoifolin and apigenin. Contrary to the findings of the present study, Duan et al. (2014) identified significantly lower levels of naringin and rhoifolin in the leaves of *C. grandis* compared with in other tissues; only 1.93 mg/g and 1.87 mg/g, respectively. This discrepancy may be attributed to differences in the ages of the leaves sampled in both studies, as it is possible that the tender leaves (20 days old) used in this study had a higher secondary metabolite content. Our results show that the fruit, tender leaves, and flowers of *C. grandis* contain high levels of flavonoids such as naringin, indicating that in addition to the fruit, Huajuhong tender leaves and flowers can also be exploited to develop novel health products, such as substitute tea. Rhoifolin, as an active substance that inhibits cancer cell growth, has attracted considerable attention in medicine (Eldahshan, 2013; Zheng et al., 2022). We observed that rhoifolin content in the tender leaves of *C. grandis* was as high as 57.70 mg/g, indicating that *C. grandis* tender leaves could be used to develop anti-cancer products.

The present study identified volatile oils such as terpenes, alcohols, fatty acids, and esters from the fruits, tender leaves, and flowers of *C. grandis*, and the results were similar to those of Xie et al. (2013). Specifically, a total of 36, 38, and 40 volatile compounds were identified in the fruitlets, petals, and tender leaves of *C. grandis*. The heat map analysis results showed that 15 volatile oils, including Myrcene, Cadinene, Terpinene, and Germacrene, are mainly present in the fruit, and these volatile oils may be the source of the aroma of *C. grandis* fruits. Ten volatile oils, including Myristaldehide, Farnesol, Nerolidol, Linalool, and Nerolidyl acetate, are mainly present in flowers, which may be related to the rich aroma of *C. grandis* flowers. In addition, 18 volatile oils, including Ocimene, Supraene, Aurapen, and Santalene, are mainly present in the leaves and are components of the unique aroma of the leaves. Most of the volatiles have important active effects (Hamdan, Mohamed & El-Shazly, 2016; Jung et al., 2018; Matuka et al., 2020), so that the volatile oils from *C. grandis* flowers and leaves have potential applications in cosmetics and healthcare industries.

To understand the potential mechanisms involved in the regulation of bioactive compound biosynthesis in *C. grandis*, we used the RNA-seq approach to investigate the transcriptome profiles in petals, fruitlets, and tender leaves. A total of nine libraries with high sequencing quality were constructed, and an average of 86.83% reads were successfully mapped to the grapefruit genome. This is significantly higher than that achieved for other grapefruit transcriptome data (Guo et al., 2015). Additionally, we mapped each library to the grapefruit genome (version 1) and identified 1,092 new transcripts, which can contribute to enriching the grapefruit genome database.

Furthermore, differential expression analysis showed that 5,605, 10,466, and 9,942 DEGs were identified in the L *vs* F, l *vs* P, and P *vs* F comparison groups, respectively. KEGG enrichment analysis of these genes showed that they were mainly enriched in metabolic pathways and biosynthetic regulation of secondary metabolites. Further analysis indicated that the pathways were involved in the synthesis of flavonoids, including “Flavonoid biosynthesis” (46 DEGs, 1.80%) and “Flavone and Flavonol biosynthesis” (6 DEGs, 0.23%). Moreover, HPLC analysis showed that there were differences in the contents of four secondary metabolites, namely naringin, rhoifolin, naringenin, and apigenin in the three tissues, indicating that DEGs may be involved in the synthesis and regulation of naringin and rhoifolin. The biosynthesis of flavonoids begins at the phenylpropane pathway (Sun et al., 2018). Phenylalanine is the first step in the phenylpropane pathway. It is converted to cinnamate under the action of PAL, and is subsequently converted to 4-coumarinyl COA under the action of CYP73A (Lucheta et al., 2007). CHS is the first enzyme in the biosynthesis of flavonoids; it converts 4-coumarinyl COA into naringin chalcone, which is further transformed into naringin by chalcone isomerase (CHI1) (Winkelshirley, 2001). Naringenin can synthesize naringin under the action of 1,2Rhat (Chen et al., 2015). The results of the present study show that the expression of key regulatory genes, such as CYP73A, CHI1, and C12RT1, was significantly upregulated in the fruitlets, indicating that these genes may be key factors regulating the biosynthesis of tangerine naringin (Chaudhary et al., 2016; Sun et al., 2018). Among them, the expression trends of *CHI1* (Cg7g005600) and *1,2Rhat* (Cg1g023820) were consistent with the naringin content trends in different tissues, indicating that it may be a key gene involved in the synthesis of naringin in *C. grandis*.

The results of the present study show that the coumarin content of the fruitlets was four times that of the petals and 20 times that of the leaves. Analysis of the coumarin biosynthesis pathway showed that the key gene *BGLU* involved in coumarin biosynthesis was differentially expressed, with *BGLU11* (Cg8g002360) and *BoGH3B* (Cg8g011340) having the highest expression levels (Table S7, Fig. 8). This result indicates that the two genes may be involved in the regulation of coumarin biosynthesis in the fruits (Wu et al., 2022).

Since results have shown that terpenoids are the main volatile compounds in *C. grandis*, we investigated the regulation mechanism of terpenoid biosynthesis in *C. grandis*. Terpenoids take isoprene C5 as the basic skeleton, and its main chain is composed of isopentenyl pyrophosphate and its isomer dimethylallyl pyrophosphate (Mani et al., 2021). Therefore, the formation of the terpenoid main chain involves a complex mechanism of two independent biosynthetic pathways. The cytoplasmic mevalonate pathway exists in most eukaryotes and some bacteria (Mahmoud & Croteau, 2002; Rodríguez-Concepción & Boronat, 2002). The methylerythritol phosphate pathway exists in plant chloroplasts, bacteria, algae, and cyanobacteria. These two pathways exist in plants and produce a series of complex chemicals. These chemicals control the development and growth of plants and interact with plants and their surrounding environment to control these interactions (Bick & Lange, 2003). KEGG analysis results showed that differential genes were mainly enriched in “Dieterpenoid biosynthesis”, “Monoterpenoid biosynthesis”, and “Fatty acid metabolism”. In addition, a total of 29 DEGs were involved in the regulation of terpenoid backbone biosynthesis. The expression levels of most differential genes in petals were significantly higher than those in the other tissues, which could be related to the strong aroma of *C. grandis* petals (Table S8, Fig. 9).

There are two main pathways of synthesis of terpenoid carbon skeletons in nature; the 2C methyl-d erythritol 4-phase (MEP) pathway and the metabolic acid (MVA) pathway, which are located in the cytoplasm and plastid, respectively (Bergman, Davis & Phillips, 2019). A total of 29 DEGs of encoding key enzymes are involved in the regulation of terpenoid backbone biosynthesis. Among these DEGs, HDR (Cg8g006150), HMGS (Cg5g009630) and GGPS (Cg1g003650) are expressed highly in the petals and may be involved in the synthesis of petal volatile oils. Monoterpenoids are C10 compounds derived from Geranyl-PP (GPP) catalyzed by geranyl pyrophosphate synthase (GPS), which can synthesize monocyclic or bicyclic compounds by cyclization (Huang et al., 2010; Mani et al., 2021). Sesquiterpenoids are C15 compounds with three isoprene (C5H8) units. They are the most diverse terpenoids and are derived from the role of Farnesyl-PP (FPP) in farnesyl pyrophosphate synthase (FPS) (Nagegowda & Gupta, 2020). In this study, a total of 45 differential genes were found to be involved in the regulation of terpenoids biosynthesis. Among terpenoids, germacrene D is the main volatile substance in the *C. grandis* cultivar, with higher content in the fruits than in the petals or leaves. Further analysis showed that nine genes encoding germacrene D synthase were differentially expressed in the tissues, with a 142- and 526-fold increase in the expression of the gene encoding germacrene D synthase (Cg2g022940) in the fruits compared with that of the leaves and petals, respectively (Table S8, Fig. 9). This result indicates that this gene may be the key regulatory gene of germacrene D biosynthesis in the fruit. Overall, these findings could serve as a reference for further studies on the regulatory mechanism of the synthesis of volatile compounds in *C. grandis*.

## Conclusions

According to targeted metabolome results, the naringin, naringenin, and coumarin contents of the fruitlets were significantly higher than those of the tender leaves and petals, whereas the tender leaves had significantly higher levels of rhoifolin and apigenin. In addition, 49 volatile oils in total were identified from three organizations, with significant differences in volatile oils among different organizations. Transcriptome analysis identified 11,514 DEGs in the fruitlets, petals, and tender leaves of *C. grandi* s. In addition, identified 9,942 genes were differentially expressed in different tissues. Further analysis showed that 20 DEGs were involved in regulation of flavonoid synthesis, 15 were involved in regulation of coumarin synthesis, and 74 were involved in the synthesis and regulation of terpenoids. CHI1 (Cg7g005600) and the 1,2Rhat gene (Cg1g023820) may be involved in the regulation of naringin synthesis in *C. grandis* fruits. HDR (Cg8g006150), HMGS, (Cg5g009630), and GGPS(Cg1g003650) could be involved in the regulation of volatile oil synthesis in *C. grandis* petals. Overall, the findings of this study contribute to the understanding of the regulatory mechanisms of the synthesis of secondary metabolites in *C. grandis*, and also provide a theoretical basis for *C. grandis* breeding.

##  Supplemental Information

10.7717/peerj.16881/supp-1 Supplemental Information 1 Raw data

## Additional Information and Declarations

### DNA Deposition

The following information was supplied regarding the deposition of DNA sequences: The RNA-Seq data are available at the Genome Sequence Archive of the Beijing Institute of Genomics Data Center: CRA006910.

### Data Availability

The following information was supplied regarding data availability: The raw measurements are available in the Supplemental Files.
